# Unraveling the Molecular Nexus between GPCRs, ERS, and EMT

**DOI:** 10.1155/2021/6655417

**Published:** 2021-03-02

**Authors:** Niti Kumari, Somrudee Reabroi, Brian J. North

**Affiliations:** ^1^Biomedical Sciences Department, Creighton University School of Medicine, Omaha, NE 68178, USA; ^2^Department of Pharmacology, Faculty of Science, Mahidol University, Bangkok 10400, Thailand

## Abstract

G protein-coupled receptors (GPCRs) represent a large family of transmembrane proteins that transduce an external stimulus into a variety of cellular responses. They play a critical role in various pathological conditions in humans, including cancer, by regulating a number of key processes involved in tumor formation and progression. The epithelial-mesenchymal transition (EMT) is a fundamental process in promoting cancer cell invasion and tumor dissemination leading to metastasis, an often intractable state of the disease. Uncontrolled proliferation and persistent metabolism of cancer cells also induce oxidative stress, hypoxia, and depletion of growth factors and nutrients. These disturbances lead to the accumulation of misfolded proteins in the endoplasmic reticulum (ER) and induce a cellular condition called ER stress (ERS) which is counteracted by activation of the unfolded protein response (UPR). Many GPCRs modulate ERS and UPR signaling via ERS sensors, IRE1*α*, PERK, and ATF6, to support cancer cell survival and inhibit cell death. By regulating downstream signaling pathways such as NF-*κ*B, MAPK/ERK, PI3K/AKT, TGF-*β*, and Wnt/*β*-catenin, GPCRs also upregulate mesenchymal transcription factors including Snail, ZEB, and Twist superfamilies which regulate cell polarity, cytoskeleton remodeling, migration, and invasion. Likewise, ERS-induced UPR upregulates gene transcription and expression of proteins related to EMT enhancing tumor aggressiveness. Though GPCRs are attractive therapeutic targets in cancer biology, much less is known about their roles in regulating ERS and EMT. Here, we will discuss the interplay in GPCR-ERS linked to the EMT process of cancer cells, with a particular focus on oncogenes and molecular signaling pathways.

## 1. Introduction

Survival and propagation of cancer cells employ highly complex cellular pathways and frequent crosstalk among them. G protein-coupled receptors (GPCRs) represent the most diverse class of surface receptor proteins that regulate a plethora of cellular functions and are utilized as drug targets for various disease conditions, including cancer [1]. They may either suppress or promote tumor growth, survival, dissemination, and metastasis through modulating multiple cellular pathways. Cancer cells experience increased protein synthesis and are exposed to a variety of stresses, such as nutrient deprivation, hypoxia, oncogenic activation, and somatic mutations leading to endoplasmic reticulum stress (ERS) due to inefficient protein folding [2]. Activation of the unfolded protein response (UPR) in response to ERS either results in an adaptive response dedicated to rectifying the protein unfolding and decreasing the load on ER or culminates into the activation of cell death pathways and autophagy, if irrecoverable. Cancer cells are known to utilize these pathways to support their growth and survival. Various studies have identified several GPCRs that play essential roles in the activation and execution of UPR. Epithelial-mesenchymal transition (EMT) is considered an important step in cancer dissemination and subsequent metastasis. GPCRs are also known to regulate EMT processes leading to metastasis.

Furthermore, ERS and UPR pathways also copromote EMT by regulating epithelial and mesenchymal marker genes. Together, GPCRs, ERS, and EMT play a pivotal role in orchestrating tumor survival and aggressiveness [3], thus providing an opportunity for therapeutic intervention. Therefore, it is important to understand the interrelationship between these processes. In this review, we discuss the roles of these three players in relation to cancer and the interaction among them. The first section of the review discusses the GPCR family and their alterations in cancer. In the second section, we will focus on the ERS and UPR pathways and how GPCRs regulate ERS. The third section is dedicated to EMT and its association with GPCR signaling. In addition, an interrelationship between ERS and EMT is also discussed.

## 2. GPCR Signaling and Cancer

### 2.1. An Introduction to the GPCR Protein Family and Signaling

The GPCRs are the largest and most diverse superfamily of receptor proteins with >1000 members predicted to be encoded by ~4% of the human genome. However, the functions of many GPCRs remain unknown [4, 5]. GPCRs transduce signals from various stimuli and regulate many biological (e.g., sensory perception, neurotransmission, embryonic development, immune response, blood pressure regulation, and homeostasis) and cellular processes (e.g., cell growth, differentiation and migration, cell apoptosis, chemotaxis, and exocytosis). Aberrant GPCR activity is implicated in many pathophysiological conditions such as neurodegeneration, cardiovascular diseases, endocrine disorders, immunological disorders, and cancer [6]. GPCRs constitute a significant portion of druggable targets, where one-third of marketed drugs are against 108 unique GPCR targets [1]. However, of the 475 FDA-approved GPCRs drugs, only nine of them have been developed for cancer indications [7].

**Figure 1:**
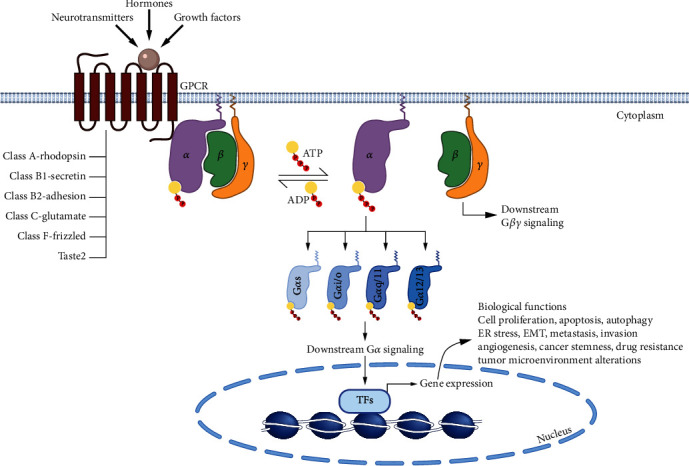
G protein-coupled receptor (GPCR) signaling pathway. Human GPCRs are seven-transmembrane receptor proteins divided into six classes (A, B1, B2, C, F, and Taste2). They receive signals from various stimuli (hormones, growth factors, neurotransmitters, etc.) and transduce the signal through G proteins in the cytosol. G proteins are made up of three subunits (*α*, *β*, and *γ*) and are anchored in the plasma membrane by binding through the *α* and *γ* subunits, while GPCRs bind G proteins through the *α* subunit. In the absence of stimuli, the G*α* subunit binds ADP and is inactive. However, upon activation, the *α* subunit binds ATP and dissociates from the *β* and *γ* subunits. There are four different types of G*α* subunits (G*α*s, G*α*i/o, G*α*q/11, and G*α*12/13), which further relay the signal to downstream targets, ultimately leading to gene transcription. The dissociated G*β*-G*γ* dimer also participates in various downstream signaling pathways. In cancer, GPCR signaling is altered, leading to the activation of genes involved in cancer cell survival and progression.

Structurally, GPCRs are made of a single polypeptide chain folded into an extracellular N-terminus, seven *α*-helical segments spanning the entire length of the membrane (hence, also known as seven-transmembrane receptors or 7TM) separated by three extracellular and three intracellular loops, and an intracellular C-terminus. Based on sequence and structural similarity, GPCRs in humans can be divided into six families: Class A (rhodopsin), Class B1 (secretin), Class B2 (adhesion), Class C (glutamate), Class F (frizzled), and Taste2 [8] (Figure 1).

GPCRs can be activated by a wide range of stimuli, including hormones, neurotransmitters, growth factors, light, and odor. In classical GPCR signaling, ligand binding induces a conformational change in the GPCR, allowing it to bind four different classes of G protein (G*α*s, G*α*i/o, G*α*q/11, and G*α*12/13). Unlike RAS, which is a single G protein subunit, the G proteins that associate with GPCRs are heterotrimeric, composed of G*α*, G*β*, and G*γ* subunits, and bound to the plasma membrane through the G*α* and G*γ* subunits. The G*α* subunit also binds to either GTP (active protein) or GDP (inactive protein); this exchange is mediated by interaction with an activated GPCR. When active, heterotrimeric G proteins dissociate into a G*α* monomer and G*β*-G*γ* dimer, which further relay the message to the downstream signaling partners [9] (Figure 1). Additional modes of GPCR activation, which mediate unique physiological or pathophysiological effects, have also been characterized as summarized by Wang et al. [10].

### 2.2. Alteration of GPCR Signaling in Cancer

The association of GPCRs with cancer was first reported in 1986 by Young and colleagues who isolated and characterized the MAS oncogene following its tumorigenicity in nude mice [11]. Since then, numerous studies have linked aberrant GPCR function with multiple cancer types. GPCRs are known to regulate a plethora of tumorigenic processes, such as cell proliferation [12], apoptosis [13], invasion [14, 15], metastasis [16, 17], angiogenesis [18], cancer stemness [19], drug resistance [20, 21], and immune suppression and regulation of tumor microenvironments [22], and are often associated with poor prognosis [23].

In various cancer types, GPCRs and their signaling pathways are known to be altered via multiple mechanisms, including elevated expression, mutations, aberrant expression of downstream G proteins, increased production of GPCR activating ligands, or aberrant expression of GRKs. Gene expression studies have revealed that many GPCRs, including orphan receptors, such as GPRC5A, show differential expression in various cancers and their subtypes (Table 1). These highly expressed GPCRs have oncogenic roles and regulate tumorigenic processes (Table 2). In contrast to expression changes, mutations in GPCRs and their consequences alone or with other genetic abnormalities in cancer have not been studied extensively. A majority of the GPCRs with frequent mutations in cancer belong to Class B2 adhesion receptors or Class C glutamate receptors. The top most mutated GPCRs among various tumor types in TCGA are GPR98, GPR112, BAI, LPHN3, GPR158, LPHN2, GRM8, GRM7, GRM3, and CELSR1. The most common mutation types found were in-frame and nonsilent mutations and are considered passenger mutations. Also, commonly mutated GPCRs (e.g., GPR98) are usually downregulated in solid tumors, while highly overexpressed GPCRs are rarely mutated. Furthermore, GPCR expression was found to be independent of driver mutations, such as in TP53 [24]. Interestingly, mutations in GPCRs are reported to either alter their basal activity or affect ligand binding or GPCR-G protein interaction or cell surface expression [25].

Aberrant activities or mutations in downstream G protein subunits, G*α*, G*β*, and G*γ*, have also been reported to promote tumorigenesis. For example, a hotspot mutation at codon 201 reduces the rate of ATP hydrolysis of GTP-G*α*, leading to constitutive expression of downstream cAMP signaling [26]. Next-generation sequencing of a total of 1348 patients with a variety of cancers revealed GNAS, GNAQ, or GNA11 aberrations in 4.1% of patients (Table 3). Also, GNAS, GNAQ, or GNA11 alterations were found to be associated with adenocarcinoma histology [27]. Similarly, GNB1 and GNB2 mutations are also associated with a variety of cancers [28], as is overexpression of GPCR ligands and alteration of GRKs (negative regulators of GPCRs) (Table 3).

## 3. GPCRs Regulate Endoplasmic Reticulum Stress (ERS)

### 3.1. Endoplasmic Reticulum Stress and UPR Signaling (IRE1, PERK, and ATF6)

Nearly one-third of all proteins destined to the endoplasmic reticulum, plasma membrane, Golgi apparatus, or lysosomes are synthesized, folded into the secondary and tertiary structures, and matured by posttranslational modification (like glycosylation) in the endoplasmic reticulum (ER) with the help of several chaperones, glycosylating enzymes, and oxidoreductases [29]. However, perturbations of ER homeostasis lead to the accumulation of unfolded proteins/misfolded proteins in the lumen of the ER, which is known as ERS. To cope with this situation, the cell activates the unfolded protein response (UPR), involving a series of signal transduction mechanisms to reduce the ER load by temporarily inhibiting global translation and refolding or degrading the accumulated unfolded/misfolded proteins.

The three critical UPR sensors in mammals are ATF6 (activating transcription factor 6), IRE1 (inositol requiring enzyme 1*α*), and PERK (protein kinase RNA-activated-like ER kinase). These three transmembrane proteins are held in an inactive state by their association with ER membrane chaperone GRP78/BIP (glucose-regulated protein 78/binding immunoglobulin protein), a member of the heat shock 70 (HSP70) family. However, the accumulation of unfolded/misfolded proteins in the ER causes the release of ATF6, IRE1, and PERK from GRP78, allowing for their activation and subsequent downstream signaling pathway induction [30]. ATF6 is a type II transmembrane protein and a member of the bZIP (basic leucine zipper) transcription factor family. The N-terminal region of ATF6 includes the bZIP domain protruding into the cytosol, while the C-terminal residues are located in the ER lumen. Upon release from GRP78, ATF6 translocates to the Golgi apparatus. In the Golgi, ATF6 is cleaved by site1 and site2 proteases (S1P/S2P) to release an active N-terminal truncated form, ATF6(n), which is then able to enter into the nucleus to initiate transcription of genes involved in protein folding, ER-associated degradation (ERAD), and apoptosis. ERAD is a process where terminally defective proteins are recognized, polyubiquitinated, translocated to the cytoplasm, and degraded by the 26S proteasome [31].

IRE1 is a type I transmembrane protein with a serine/threonine kinase domain and an endoribonuclease domain located on the cytosolic side of the protein. Unlike ATF6, dissociation from GRP78 leads to di/oligomerization and transphosphorylation of IRE1, inducing a conformational change activating its endoribonuclease activity. The latter catalyzes X box protein-1 (XBP1) mRNA splicing to produce XBP1s (spliced XBP1), a bZIP transcription factor. XBP1s activates the expression of genes involved in protein folding, ERAD trafficking, lipogenesis, and inflammation. To reduce the protein load in the ER, IRE1-dependent decay (RIDD) is also activated [32]. IRE1 can also induce the tumor necrosis factor receptor- (TNFR-) associated factor 2 (TRAF2)/apoptosis signal-regulating kinase 1 (ASK1)/JNK cascade, which also contributes to activation of cell death [33].

Similar to IRE1, PERK is also a type I transmembrane protein with a serine/threonine kinase domain. When unbound from GRP78, PERK also undergoes dimerization and transphosphorylation to activate its kinase domain. The activated kinase then inhibits eukaryotic initiation factor 2*α* (eIF2*α*) by its phosphorylation at serine-51, thus shutting down global protein translation. However, it selectively increases levels of transcription factor ATF4, which induces amino acid biosynthesis and phosphorylates Nrf2, thereby controlling the antioxidant response. One of the direct targets of ATF4 is C/EBP homologous protein (CHOP), which activates genes responsible for mitochondrial-mediated apoptosis. CHOP and ATF4 regulate several autophagy-related genes, p62, Atg5, Atg7, and Atg10 [34]. CHOP also activates DNA damage-inducible protein (GADD34) (a regulatory subunit of protein phosphatase 1) to dephosphorylate eIF2*α* and resume protein translation and limit PERK signaling [35].

Under conditions of prolonged and unmitigated ERS, all three arms of UPR can lead to induction of lysosome-mediated autophagy through various mechanisms, including the IRE1-JNK-Bcl-2, PERK-eIF2*α*-ATF4, or ATF6-XBP1-Atg axes [36–38].

## 4. ERS in Cancer

### 4.1. ERS Inducers in Cancer: Oxidative Stress, Hypoxia, Nutrient Starvation, Low pH, and Oncogenes

Tumor cells evade normal cell cycle and cell death regulatory processes, facilitated by inactivation of various tumor suppressors (e.g., p53 and PTEN), while the gain of oncogenic capabilities by others (e.g., MYC and RAS) increased proliferation rates compared to normal cells. To sustain such high proliferation rates, an induction of global protein synthesis is required. This increases the burden on the ER as protein synthesis often exceeds the capacity of ER folding machinery, leading to the accumulation of unfolded proteins and the induction of ERS [36]. These increased growth rates require high metabolic rates in cancer cells leading to increased consumption and depletion of nutrients such as glucose and amino acids from the microenvironment [2]. As both are required for ER functions, de novo protein synthesis, and posttranslational glycosylation of proteins, their restricted supply hinders ER functions leading to ERS. Tumor cells also rapidly consume oxygen in their vicinity which cannot be readily replenished due to insufficient vascularization, leading to the low oxygen levels in the initial tumor microenvironment, known as hypoxia. Hypoxia also contributes to further ERS. Two major protein modifications in the ER are disulfide bond formation and N-linked glycosylation, and while disulfide bond formation is an oxygen-independent process, posttranslational folding is oxygen-dependent [39]. Thus, the absence of oxygen leads to protein unfolding, inducing further ERS. Protein overload conditions also result in reactive oxygen species (ROS) generation due to the oxidative folding process. Increased ROS targets ER-resident enzymes, proteins, and calcium channels, leading to the release of Ca^2+^ into the cytosol [40]. Furthermore, to meet the rapid energy/ATP demands by fast-growing tumor cells, they enhance their glucose uptake and shift their metabolism from oxidative phosphorylation to glycolysis despite the presence of oxygen. Anaerobic oxidation of glucose produces abundant lactic acid levels, which are excreted into the surrounding microenvironment increasing the acidity. The extracellular microenvironment of tumors becomes acidic, ranging from pH 6.5 to 6.9, and acidosis is also known to induce ERS pathways [41].

Activated oncogenes, like MYC, RAS, and BRAF, also trigger UPR. N-MYC and c-MYC are potent oncogenes that increase global protein synthesis, thereby inducing the PERK/eIF2*α*/ATF4 branch of UPR to support cell survival by cytoprotective autophagy. While c-MYC was shown to induce the IRE1-XBP1s pathway in a triple-negative breast cancer model, UPR/XBP1s can also directly activate MYC expression [42]. Oncogenic BRAF V600E also induces ERS by interaction with GRP78 and induction of cytoprotective autophagy [43] and IRE1/ASK1/JNK-mediated apoptotic response [44]. Mutant p53 has also been shown to induce ATF6 activation to support cell survival and inhibition of proapoptotic JNK and CHOP [45]. Oncogenic G12V mutation in H-RAS activates the prosurvival PERK and XBP1 branches of UPR to support cell growth [46].

### 4.2. Crosstalk between UPR and Cell Signaling Pathways in Cancer

Bidirectional crosstalk between UPR and other cell signaling pathways at multiple levels fine-tunes cellular stress responses in cancer. Previous studies have revealed that cancer cells utilize UPR to promote growth and survival in hostile tumor microenvironments. This involves the regulation of cell survival and growth pathways (PI3K/AKT/mTOR, p38, and RAS/RAF/MEK/ERK) as well as cell death/apoptosis pathways (TRAF2/JNK cascade, mitochondrial-mediated cell apoptosis) via activating the UPR.

#### 4.2.1. NF-*κ*B and UPR

NF-*κ*B is a transcription factor and controls many genes involved in promoting tumorigenesis [47]. A study by Tam and colleagues suggests that the PERK and IRE1 arms of the UPR are crucial regulators of NF-*κ*B signaling, and contribution by both pathways is required to maintain the full activity of NF-*κ*B during ERS. IRE1 interaction with TRAF2 and tumor necrosis factor-alpha (TNF-alpha) activates JNK/AKT, which phosphorylates I*κ*B kinase *β* (IKK), thus maintaining a basal level of I*κ*B kinase *β* (IKK) activity. IKK phosphorylates the inhibitor of NF-*κ*B (I*κ*B), which promotes its ubiquitination and subsequent degradation resulting in stabilization of NF-*κ*B. Furthermore, PERK-mediated inhibition of global translation reduces I*κ*B, thus contributing to full activation of the NF-*κ*B pathway [48].

#### 4.2.2. MAPK Signaling and UPR

The UPR modulates all three axes of MAPK signaling, including JNK, p38, and ERK1/2. IRE dimerization leads to its association with TRAF2 and ASK1, which can phosphorylate MKK4/7 leading to activation of JNK [33]. While JNK is often considered proapoptotic, JNK mediated phosphorylation of c-Jun, and subsequent transcription of Adpt78 promotes cell survival [49]. ER stress-mediated activation of the IRE/JNK axis also regulates protective autophagy by interaction with Beclin-1 [36–38]. ASK1 can also activate MKK3/6 leading to activation of p38 which leads to both p38-dependent induction of ATF6 expression and also phosphorylation and activation of CHOP to promote its apoptotic functions [50]. Furthermore, IRE1 also activates ERK1/2 prosurvival signaling [49], and inhibition of the MEK/ERK pathway by U0126 has been shown to sensitize breast cancer cells to ER-induced apoptosis [51].

#### 4.2.3. PI3K/AKT Signaling and UPR

**Figure 2:**
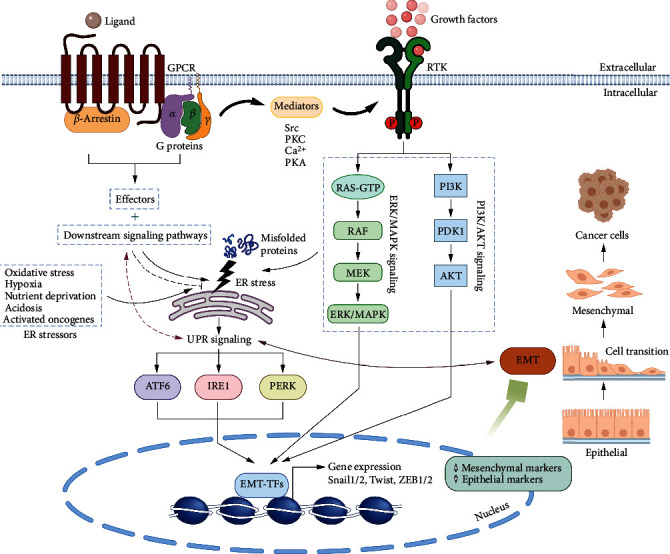
Crosstalk of GPCR signaling and ERS during EMT. Activation of GPCRs and ERS facilitates the EMT process of cancer cells. Upon ligand binding, GPCRs transmit signals via heterotrimeric G proteins, *β*-arrestins, and crosstalk with receptor tyrosine kinases (RTKs) through signaling mediators. The activation of RTKs by growth factors drives EMT through ERK/MAPK and PI3K/AKT signaling cascades which in turn lead to induction of ERS. GPCRs controlling downstream effectors and multiple signaling pathways regulate ERS by interacting with IRE1, PERK, and ATF6 arms of the UPR. The UPR signaling is stimulated by ER stressors including oxidative stress, hypoxia, nutrient deprivation, acidosis, and activated oncogenes. Bidirectional crosstalk between UPR and cell signaling pathways refines cellular stress responses (dotted double arrow). GPCRs can either activate (curved arrow) or inhibit (curved dotted line) ERS. GPCR-ERS-induced UPR signaling pathways concurrently induce EMT during tumorigenesis. Activation of EMT occurs in response to GPCR-mediated signaling and ERS by upregulation of EMT-TFs (Snail1/2, Twist, and ZEB1/2), accompanied by an increase of mesenchymal markers and a decrease of epithelial markers. Reciprocal regulation between EMT and UPR signaling is observed during tumor progression (curved double arrow).

Various reports highlight a potential role of PI3K/AKT signaling upstream as well as downstream of UPR in a context-dependent manner. Recently, Winnay et al. observed that inhibition of the PI3K/AKT pathway results in reduced phosphorylation of PERK at Thr-980, leading to a reduction in expression of ATF4 and CHOP as well as a decrease in phosphorylated IRE1, BIP, and XBP1s in tunicamycin-treated brown preadipocytes, hepa1c1c7 mouse hepatoma cells, and mouse embryonic fibroblasts, suggesting a positive role of this pathway in induction of ERS [52]. The PI3K/AKT pathway was also shown to positively regulate the UPR in bleomycin-inducing pulmonary fibrosis [53]. However, according to another study by Mounir et al., PI3/AKT activation leads to Thr-799 phosphorylation-mediated inactivation of PERK and downstream eIF2*α* in glioblastoma U87 cells, human breast cancer SkBr3 cells, and spontaneously immortalized mouse embryonic fibroblasts (MEFs), thereby inhibiting their protective effect to tumor cells [54]. Crosstalk between PI3K/AKT and MEK/ERK signaling under ERS conditions has also been observed. AKT was found to phosphorylate c-RAF on Ser-259, and during ERS in HCC cells (HEP3B and SMMC-7721), AKT activity was suppressed, and a consequent increase in the MEK/ERK pathway was observed to support cell proliferation [55]. In another study, PERK was shown as a direct target of AKT, leading to its activation during hypoxia [56]. Induction of ERS/PERK was shown to induce cytotoxic autophagy via inhibition of the AKT/TSC/mTOR pathway [57]. In hormone therapy-resistant breast and prostate cancer cells, GRP78 was actively found to be translocated to the cell surface, where it interacts with PI3K leading to activation of the proproliferative PI3K/AKT pathway [58]. Therefore, there is important crosstalk between these two critical oncogenic pathways and regulation of ERS (Figure 2).

#### 4.2.4. TGF-*β* and UPR

TGF-*β* is known to play a role in the epithelial-mesenchymal transition (EMT), cell migration, and invasion. Downregulation of TGF-*β* in different cancer cell lines promoted cell death through ERS via enhancing the ASK1/JNK axis [59]. TGF-*β* is also induced as a downstream effector of the UPR via MAPK signaling to regulate the EMT process [60]. Both ATF6 and XBP1s can upregulate the expression of TGF-*β* [61], further defining a role for TGF-*β* in the UPR.

#### 4.2.5. Wnt/*β*-Catenin Pathway and UPR

Activation of the Wnt/*β*-catenin pathway by CP21R7 has been shown to induce the IRE1-mediated increase in the expression of genes involved in metabolism, insulin resistance, and lipogenesis to promote cell survival [62]. Furthermore, in multiple myeloma, accumulation of *β*-catenin resulted in induction of cell cycle arrest via CHOP/p21 activation and apoptosis via c-Jun/p73 induction. However, whether these pathways are interrelated or independent has yet to be established [63].

### 4.3. GPCRs and ERS Pathways in Cancer

Numerous studies suggest a complex relationship between GPCRs and ERS, as both play critical roles in regulating one another. Many GPCRs are known to act upstream of ER-UPR as sensors of various stress signals in cancer and other pathological conditions. They may interact either directly or indirectly with downstream ATF6, IRE1, and PERK arms of the UPR to induce a specific response. The GPCR signaling cascade culminates into the cell-protective or apoptotic response by UPR to promote cancer cell survival. The UPR may also be activated in response to mutant GPCRs. For instance, mutations that allow smoothened activation in the absence of ligands may induce the UPR via activation of XBP1 signaling [64]. On the other hand, UPR is also known to modulate downstream GPCRs to benefit the survival of cancer cells.

In response to microenvironmental stress signals in cancer cells, activation of some oncogenic GPCRs regulates UPR to inhibit the apoptotic/cell death pathways to promote cancer cell survival. For example, overexpression of CXC chemokine receptor 4 (chemokine receptor family) (CXCR4) of the rhodopsin family is induced in response to various stresses, including serum deprivation, hypoxia, and contact inhibition [65], and associated with metastasis of cancer cells [65–67]. Its downregulation sensitizes osteosarcoma cells to apoptosis with significant upregulation of ERS markers, GRP78, XBP1, and p-eIF2*α*/CHOP, and inhibition of the PI3K/AKT/NF-*κ*B signaling axis [68]. Another member of the same family, lysophosphatidic acid receptor (LPAR), is known to regulate a variety of tumorigenic functions including proliferation, survival, angiogenesis, invasion, and metastasis [69] and is implicated in inhibiting ER stress-mediated apoptosis in mesenchymal cells by inhibition of p38 activation via the LPA1/3-Gi/ERK1/2/MAPK1 signaling axis under the conditions of hypoxia and serum deprivation [70]. The ERS-mediated protective effect was also observed in oligodendrocyte precursor cells upon induction of LPA1/3 receptors via modulation of UPR proteins BIP, GRP94, CHOP, and XBP1s [71]. In another example, prostaglandin receptor EP2 (Class A, lipid receptor family) activation was shown to protect against ER stress-induced apoptosis via cAMP-mediated downregulation of p53 and its target gene PUMA [72]. Elevated EP2 signaling is associated with breast cancer [73], cervical cancer [74], and bladder cancer [75]. High expression of AGTR1 is also associated with metastasis of multiple cancer types and is negatively correlated with the prognosis of ovarian cancer. High levels of expression have also been observed in breast, skin, ovary, cervical, and prostate cancers. Angiotensin II stimulation of multicellular ovarian spheroids resulted in proliferation and migration by induction of ERK1/2 and AKT pathways further suppressing the ERS pathway and consequently inhibition of JNK signaling and extrinsic cell apoptosis pathways [76]. Among the secretin family of GPCRs, the glucagon-like peptide-1 receptor (GLP1R) exerts its antiapoptotic effect in chondrocytes by activating the PI3K/AKT and NF-*κ*B pathways and suppressing the ERS response [77]. On the other hand, in beta cells, GLP1R was observed to induce ERS by upregulation of PERK/ATF4 and IRE1/XBP1s [78] signaling and Bcl-2 and X-chromosome-linked inhibitor of apoptosis as well as inactivation of caspase 12 [79]. In addition, the Class F frizzled receptor, *β*-catenin, and Wnt/*β*-catenin pathway negatively regulate XBP1-mediated HIF1*α*-directed gene expression in the RKO colon cancer cell line to promote cell survival. However, under hypoxic conditions, the UPR is activated in RKO cells, which reduces the stability of *β*-catenin via reduction of LRP6, a *β*-catenin coreceptor [80]. The above studies demonstrate a pivotal role of GPCRs in response to specific environmental cues in reprogramming the ERS pathway to inhibit the initiation of the apoptotic response by the UPR. Blocking the activation of such GPCRs by antagonists is therefore a potential strategy in cancer intervention.

Some oncogenic GPCRs can also regulate ER-induced autophagy. The S1PR family of receptors includes five receptors S1PR1-S1PR5 and binds to sphingosine-1-phosphate (S1P) as their ligand. Studies have shown that activation of S1PR5 by S1P induces ERS pathways involving ATF6, PERK, and IRE branches, upregulating ROS production and inducing autophagy to enhance cell survival in prostate cancer cells [81]. Similarly, one of the primary GPCR sensors of an acidic environment, GPR68 (OGR1), exerts survival benefits via regulation of autophagy. GPR68 is overexpressed in numerous tumor types, including pancreatic ductal adenocarcinoma (PDAC), cervical squamous cell carcinoma, endocervical adenocarcinoma (CESC), breast adenocarcinoma, and ovarian cancer. Tan and colleagues demonstrated through live-cell imaging that GPR68 is localized on the plasma membrane in mildly acidic extracellular conditions but is internalized in slightly basic conditions [82]. GPR68 activates ERS in an intestinal epithelial cell model via the IRE1/JNK pathway and inhibits late-stage autophagy to promote cell survival [83].

**Figure 3:**
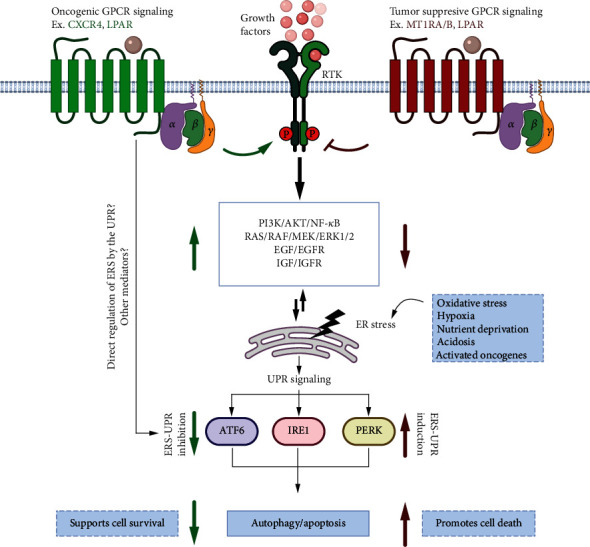
GPCR-ERS-UPR in cancer. Overexpression of the GPCRs with oncogenic roles (e.g., CXCR4 and LPAR, green) in the conditions of hypoxia, nutrient deprivation, ROS, activated oncogenes, or acidic environment is associated with activation of survival pathways (PI3K/AKT/NF-*κ*B, MAPK, and growth factor mediated signaling) to support cancer cell survival. At the same time, via regulation of IRE, ATF6, and PERK, GPCRs can inhibit cell death pathways, apoptosis, and cytotoxic autophagy (signaling indicated with green arrows). On the other hand, GPCRs with anticancer roles (MT1RA/B and CNR1/2, red) are associated with activation of the ERS-mediated UPR signaling to induce apoptosis and autophagy-related cell killing (signaling indicated with red arrows). Complex bidirectional crosstalk between UPR pathways and various cell survival and growth signaling pathways is involved in mediating the GPCR-related cancer cell fate. However, the exact sequence of this crosstalk for a given GPCR or other possible mediators still needs to be explored.

On the contrary, some GPCRs have antitumor functions and utilize UPR machinery to mount a cytotoxic response. Melatonin treatment was proposed as a possible cancer therapy as it mediates anticancer effects through melatonin GPCRs, MT1RA and MT1RB [84, 85]. It has been shown to activate ERS and induce apoptosis in a diethylnitrosamine-induced hepatocarcinogenesis mouse model [86, 87]. Melatonin was also shown to sensitize human hepatoma cells to ERS-induced apoptosis [88] and to decrease cell proliferation in the hepatocarcinoma HepG2 cell line via melatonin receptor 1, MT1RA, and decrease cAMP and ERK signaling [89]. In a canine breast cancer model, melatonin induced apoptosis and inhibition of tumor growth in estrogen receptor- (ER-) positive tumors with high MT1RA expression [90] and metastasis in triple-negative breast cancer cells [84]. The cannabinoid receptor family members, CNR1 and CNR2, are also implicated in regulating ERS. High expression of CNR2 in breast cancer is associated with inhibition of EGF/EGFR and IGF-I/IGF-IR pathways and a better prognosis [91]. Greenhough and colleagues reported that induction of CNR1 and CNR2 in colorectal cancer cells inhibits tumorigenic RAS/MAPK and PI3K/AKT signaling [92], while the loss of CNR1 in tumor samples from colon cancer patients correlated with tumor growth [93]. Furthermore, hypoxic conditions decreased expression of CNR1 and CNR2 in rat glial cells, and low expression of CNR2 in CRC patients is associated with poor prognosis [94]. In contrast, low expression of CNR1 accelerates intestinal tumor growth [93]. Shrivastava and colleagues showed that induction of CNR1 in breast cancer cells leads to the induction of ERS, which promotes autophagy and apoptosis and inhibition of AKT/mTOR/4EBP1 signaling [95]. G protein-coupled estrogen receptor (GPER) has been demonstrated to have an antitumor role by inducing ER-mediated cell death via activation of the PERK, ATF6, and IRE1 branches of the UPR [96, 97]. Among the secretin glucagon family of receptors, GHRH and GHRH receptor signaling were shown to be involved in apoptosis in JEG-3 cells via activation of AKT and eIF2*α* [98]. These studies hint towards another contrasting role of GPCRs in cancer, by mediating cytotoxic effects on cancer cells which can be exploited by the use of agonists against this class of GPCRs for clinical intervention. A general example of crosstalk between GPCR signaling and ERS-UPR pathways is depicted in Figure 3.

Moreover, not only do GPCRs regulate UPR, but UPR can also activate or inactivate downstream target GPCRs to achieve a particular response. Studies suggest that oncogenic GPCRs are activated in response to ERS and the UPR. Roberts et al. suggested a link between EP2 and ERS in colon cancer cells in response to glucose deprivation. According to their model, the absence of glucose leads to increased extracellular EP2 by stimulating PI3K/AKT/Cox2 signaling and downregulation of the EP2-degrading enzyme, 15-PGDH, via UPR/CHOP-mediated degradation to support tumor survival [99]. Cancer cells can also exploit the UPR to downregulate tumor-suppressive GPCRs. GABA_B_ receptors are antitumorigenic in multiple cancer cells, including the pancreas, liver, lung, colon, and breast, and suppress tumor growth *in vivo* [100]. Low oxygen and glucose conditions leading to ERS/CHOP activation were shown to downregulate the cell surface expression of GABA_B_ in neurons [101].

Despite the complex nature of the intracellular signaling of the GPCR-ERS-UPR pathway and its limited understanding in oncology, their crosstalk opens the opportunities to develop alternative anticancer therapies through many approaches. It is well known that the ERS pathway is one of the major determinants of cellular health and survival or its demise, but it is also a major mechanism hijacked by cancer for sustaining its survival and growth. As discussed in this section, some cancer-favorable GPCRs are associated with blocking ERS-UPR-induced apoptosis and are upregulated in cancer, while other cancer-antagonistic GPCRs are associated with induction of cell death mechanisms and are often mutated and/or downregulated. As indicated by various mRNA expression-based studies, every tumor type shows the upregulation of numerous GPCRs. These upregulated GPCRs can be an easy target for cancer therapy by blocking their activation via small molecule antagonists to inhibit oncogenic GPCR signaling. Therefore, it would be interesting to inhibit GPCR-induced UPR branches that are activated in response to increased cell survival pathways following ERS induction by ER stressors. Through the activation of oncogenes, a sustained stimulation of GPCRs and RTK receptors is observed in certain tumors that can also trigger the UPR to promote tumor growth and survival. Thus, suppressing ligand binding and the activation of these receptors with pharmacological antagonists or small molecule inhibitors should be able to quench induction of UPR pathways regulating tumor formation and adaptation.

Inhibiting the interaction between a GPCR and its ligand could be a useful strategy in cancer. Additionally, to induce or enhance cytotoxic signaling, chemical or peptide-based agonists are also being considered. Simultaneously combining agents that can accelerate severe ERS to induce cell death along with specific inhibitors of growth factor receptors could serve as another effective strategy exploiting GPCR-ERS crosstalk to prevent tumor progression. Currently, only nine GPCRs have FDA-approved drugs for cancer. However, with the recent advancements in technologies such as RNA-seq and Crispr-cas9, more information about GPCR deregulation in cancer is becoming apparent. With this information, some novel GPCR targets in cancer that already have been approved for other ailments might prove useful in cancer and be candidates for repurposing. However, currently, there are many challenges to utilizing GPCRs as successful therapeutic targets. While mRNA expression data is available for various tumor types, proteomic analysis for a majority of GPCRs in cancer is lacking. Due to their large structure and hydrophobic nature, they are difficult to isolate and crystallize. Most of the structure prediction has been done via homology modeling. In addition, many GPCRs found upregulated in cancer are orphan receptors, and thus, their ligands, biology, and/or pharmacology are unknown. Therefore, identifying ligands for orphan receptors, especially those that have altered expression or correlations with patient survival, should be a focus of futures studies as they could provide important information in our knowledge of cancer biology and serve as novel therapeutic targets.

Our knowledge of the functions of GPCRs in relation to cancer remains limited. Moreover, to target GPCR-ERS-UPR pathways in cancer, it is important to first identify the critical GPCR regulators of the ERS-UPR pathway during tumorigenesis as there are limited studies exploring a GPCR-ERS relationship in cancer, with most assessing these interactions in the context of other diseases. Various studies imply that GPCRs and ERS pathways crosstalk in a manner such that they play a significant role in regulating one another; hence, a thorough understanding of the relationship between these pathways is necessary with respect to cancer types to fully exploit potential opportunities for therapeutic intervention. In addition, further studies are required to understand how specific GPCRs in various cancer types direct the execution of ERS-UPR. Is it prosurvival or proapoptotic? What are the downstream pathways involved and potential crosstalk with other cancer-related pathways, and if any, are other mediators involved? It would also be important to know the specificity of ligands to GPCRs and also GPCR specificity to downstream interacting partners to avoid adverse effects.

## 5. GPCRs Regulate EMT

### 5.1. The Process of EMT in Cancer

The epithelial-mesenchymal transition (EMT) is a highly dynamic process in which polarized epithelial cells change their characteristics into a mesenchymal phenotype [102] and occurs during implantation, normal embryogenesis, organ development, organ fibrosis, tissue regeneration, and wound healing [103, 104]. The EMT process involves the loss of cell-cell adhesion, apical-basal polarity, degradation of the basement membrane and extracellular matrix (ECM), remodeling of the cytoskeleton, and changes in expression of cellular markers. In cancer, EMT has been shown to play crucial roles in multiple steps of tumorigenesis, including tumor proliferation, migration, invasion, metastatic expansion, and resistance to cancer therapies [105]. It is also known that EMT is a binary process through the so-called mesenchymal-epithelial transition (MET) by which the acquired mesenchymal features of a cell population can be reversed into epithelial characteristics [103]. Though the fully underlying mechanisms for plasticity between EMT and MET are poorly understood, the MET has been shown to occur when a transcriptional program of EMT-TFs is inactivated, and upon surviving, circulating cancer cells move across the basement membrane to reach a desirable metastatic niche of distant sites to generate secondary tumors [106–108]. EMT is commonly defined as the loss of the epithelial marker E-cadherin and the expression of the mesenchymal marker vimentin. However, transition in phenotypes between the epithelial and mesenchymal cells is much more complex and involves distinct molecular processes and various markers to complete the transition states. These include induction of the expression of specific proteins at cell junctions, reorganization of cytoskeletal structures, upregulation of ECM-degrading enzymes, and activation of transcriptional regulatory networks and EMT-activating transcription factors (EMT-TFs) Twist, Snail1 and Snail2 (also known as Slug), several other basic helix-loop-helix (bHLH) transcription factors such as zinc finger E-box-binding homeobox 1 (ZEB1) and ZEB2, and well-known mesenchymal markers, vimentin and N-cadherin [109] (Figure 2). Changes in the expression of noncoding RNAs such as microRNAs and long noncoding RNAs have also been observed to participate in regulating the EMT [110, 111]. Likewise, the EMT is coordinated by epigenetic regulation, chromatin remodeling, alternative splicing, posttranslational modifications, stabilization, and altered subcellular localization of proteins [112]. These signals acting through biochemical mechanisms accelerate the epithelial cells to undergo phenotypic changes and cause excessive proliferation and acquired invasiveness.

As a consequence of the EMT, cancer cells are eventually able to invade, extravasate, and initiate metastatic dissemination to distant organs, contributing to cancer progression in advanced stages and poor prognosis for patients. Induction of EMT is complex and appears to be stimulated by inflammation, ROS, hypoxia, cytokines, growth factors secreted from the tumor microenvironment and stroma, metabolic changes, immune responses, and anticancer agents. As such, many inducing factors and a variety of intracellular signaling pathways, like PI3K/AKT, NF-*κ*B, and MAPK/ERK, concurrently orchestrate the complex EMT process. However, it is still unclear what are the major upstream regulators of these signaling networks to induce EMT in tumor cells. Notably, a pivotal player controlling the transduction of these multiple pathways may be associated with GPCRs. Deciphering the crosstalk between GPCRs and molecular signaling pathways leading to activation of EMT during tumorigenesis might provide new insights into the molecular events converting epithelial cells into mesenchymal cells with stem cell-like characteristics and pave the ways for developing possible therapeutic interventions.

### 5.2. GPCRs Regulate the EMT Process

Activation of EMT results from the induction of transcription factors involved in cell adhesion, cytoskeleton remodeling, migration, and invasion. Expression and transactivation of EMT-related genes occur in response to intracellular signaling pathways. It is well accepted that the activation of GPCRs is a critical mechanism required for facilitating EMT and tumor progression.

The constitutive activation of GPCRs in tumors is stimulated by overexpression of the receptors and increases in the release or production of their ligands [113]. Upon activation, GPCRs trigger EMT and tumor progression by crosstalk with growth factor receptors, G proteins, tyrosine kinases, LPA-mediated signaling, and other oncogenic signaling pathways [114, 115]. The most prominent growth factor receptor playing an integral role with GPCRs is the epidermal growth factor receptor (EGFR), regulating tumor growth, invasion, and progression in various human cancers [116, 117]. Other GPCR ligands such as LPA, prostaglandins, bradykinin, gastrin-releasing peptide (GRP), and bombesin (BN) can also transactivate EGFR to induce cancer proliferation, survival, and invasion. The critical signaling intermediates involved in GPCR-EGFR crosstalk include Src, PI3K/AKT, PDK1, MMP, and ADAMs [117–119]. For involvement of chemokine activation, CXCL12 or EGF has been observed to upregulate CXC chemokine receptor 4 (CXCR4) and EGFR to cooperatively increase gastric cancer cell migration. Both ligands can induce the activation of IKK*αβ* and p65 of the NF-*κ*B pathway to promote metastasis [120].

In addition, GPCRs transmit signals via heterotrimeric G proteins with different G protein subunits to promote cancer cell migration, invasion, and tumor dissemination [121, 122]. To determine the biological significance of G protein-dependent signaling pathways, specific G protein inhibitors have recently been developed [123]. For instance, Kirui et al. identified that inhibition of G*βγ* signaling by using a G*βγ* inhibitor (M119K) and a G*βγ*-sequestering peptide (*β*ARK1ct) suppressed migration and invasion of breast cancer cells MDA-MB-231 and MDA-MB-436 cultured in NIH-3T3-conditioned media. Both compounds reduced lamellipodium formation, a key process of metastasis through Rac-dependent signaling [124]. Another major downstream effector of GPCR signaling is PI3K, in which both PI3K*β* and PI3K*γ* are mediated though G*βγ* subunits [125, 126]. PIP3/PI3K is overexpressed in solid tumors, where it promotes metastasis. Activating GPCRs, particularly through PI3K*β*-mediated G*βγ* binding, plays a crucial role in breast tumorigenesis. A recent study identified a role for GPCRs in promoting metastasis by acting on PI3K signaling pathways [125]. A mutation of p110*β*, a subunit of Class I PI3K, that disrupted G*βγ* binding was found to markedly inhibit invadopodium-mediated ECM degradation, breast tumor extravasation, and metastasis with moderate reduction of cell migration. Disruption of p110*β*-G*βγ* binding could constitute a novel therapeutic pharmacological approach to preventing metastasis in breast cancer patients.

Recently, a family of adaptor proteins called *β*-arrestins has been described as a scaffolding protein for GPCRs to control signal transduction and drive cancer progression, mainly by affecting invasion and metastatic potential of cancer cells via various signaling pathways [127, 128]. Mechanistically, *β*-arrestins control cell migration by inducing the expression and localization of proteins associated with remodeling of the actin cytoskeleton at the leading edge of cells. The related molecules that connect GPCR-*β*-arrestins and cell migration include filamin, cofilin, c-Src, and small monomeric GTPases. *β*-Arrestins, interacting with EGFR, Rho-GEFs, or other signaling pathways, also regulate cell migration, invasion, and metastasis by inducing transcription of EMT-related genes, increasing activity of ECM-degrading enzymes, and promoting invadopodium formation [128, 129].

Interestingly, some GPCRs may exert their roles in switching between EMT-MET processes. For example, overexpression of GPCR19, an orphan GPCR, drives MDA-MB-231 breast carcinoma cells from a mesenchymal phenotype towards an epithelial phenotype [14]. The upregulation of E-cadherin and a decrease in invasion and migration and reduction in stress fibers were observed in GPCR19-overexpressing cells. Furthermore, activation of the GPCR19 with a novel ligand, adropin, further increases E-cadherin expression dependent on MAPK/ERK signaling. It is interesting to note that MET is known to promote secondary tumor outgrowth [103, 130]; therefore, it is interesting to speculate that GPCR19 might play a role in the colonization of metastatic breast tumors.

Based on these important studies, preventing EMT and tumor progression by targeting GPCRs with pharmacological manipulation may serve as a promising therapeutic intervention for cancer patients. Given that GPCRs share crosstalk with multiple pathways, this may provide an opportunity to develop combination therapeutic strategies modulating GPCR function and interacting molecules with select inhibitors which may increase therapeutic efficacy while minimizing adverse side effects.

## 6. Relationship between ERS and EMT

### 6.1. Effects of ERS on EMT: EMT Inducers ZEB, Snail, Twist, N-Cadherin, and E-Cadherin

EMT is often characterized by the upregulation of mesenchymal transcription factors including Snail, ZEB, and Twist superfamilies, loss of the epithelial marker E-cadherin, adherens junction proteins, and cell polarity, and a rearrangement of the cytoskeleton to display a spindle-shaped morphology with increased ability to migrate and invade surrounding tissues and ECM. Tumor cells can increase invasiveness and metastasis by activating EMT during their development [104]. Characteristic features of cancer cells undergoing EMT share similarities to what is observed in cancer stem cells [131]. This suggests that, in the absence of self-renewal and the capacity to differentiate, cells undergoing EMT obtain mesenchymal traits and aggressive properties, often leading to resistance to effective cancer treatments.

EMT has a critical role in tumor progression by inducing tumor invasion and metastasis [132], and EMT-TFs, including Snail, ZEB, and Twist superfamilies, are important in regulating EMT states. The expression patterns of EMT-TFs are varied in different human carcinomas and are similarly involved in EMT plasticity and maintaining the migratory phenotype. Among these transcription factors, Snail has a prominent role as an inducer of EMT in primary tumors by repressing the *CHD1* gene encoding E-cadherin [133]. While our mechanistic understanding at the levels of epigenetic modifications and posttranscriptional control in the regulation of EMT-TFs have been extensively studied, upstream signaling pathways regulating the expression of EMT-TFs are complex and require further elucidation.

A recently identified hallmark of carcinogenesis is ERS, which has been proposed as an additional mechanism regulating EMT activation [134]. UPR signaling induced by ERS may promote EMT in various cancers and may represent a new target for the treatment of solid and hematopoietic tumors as it controls multiple steps of malignant progression, including EMT [132]. In breast cancer, XBP1 has been identified as a novel regulator of EMT and cancer progression. A high level of XBP1 in primary and metastatic breast tumors is correlated with tumor stage and poor prognosis of patients [135]. Overexpression of XBP1 was shown to parallel the increased expression of mesenchymal markers N-cadherin and vimentin but negatively correlated with E-cadherin expression. In contrast, the knockdown of XBP1 restored expression of E-cadherin and cell-cell junction formation, inhibiting breast cancer cell invasion and tumor formation. This finding also demonstrates that XBP1 promotes EMT and cell invasion through upregulation of Snail gene expression. In glioblastoma multiforme (GBM), the modulation of IRE1 on tumor properties has been investigated in various GBM primary cell lines [136]. Overexpression of IRE1 promoted cell migration, increased the expression of EMT genes vimentin, ZEB1, and TGF-*β*2 and chemokine genes CXCL2, CCL2, and IL-6, and promoted immune cell infiltration. These studies confirm the contribution of IRE1/XBPs signaling as a critical mechanism linked to EMT and tumor aggressiveness phenotypes.

UPR and EMT markers are commonly observed to be upregulated under ERS conditions in different human tumors. The sustained hypoxia found in rapidly growing tumors can activate UPR to promote adaptation to low oxygen supply and maintain cell survival [137, 138]. In gastric cancer cells, UPR-related proteins PERK, ATF4, and ATF6 are upregulated by severe hypoxia (0.1% O_2_) but not under normoxia or mild hypoxia conditions [139]. In breast cancer, the PERK branch of the UPR increases cell migration upon hypoxic induction through ATF4-mediated induction of lysosomal-associated membrane protein 3 (LAMP3), a lysosomal protein that is also relevant to cancer metastasis [140, 141]. The metastatic role of LAMP3 was also further confirmed in head and neck squamous cell carcinoma [142], oral squamous cell carcinoma (OSCC) [143], and hepatocellular carcinoma (HCC) [144] and has recently been reported as a direct target of ATF4 [145]. Prolonged ERS resulted in irreversible EMT in human peritoneal mesothelial cells (HPMCs), accompanied by activation of the Smad2/3 pathway, nuclear translocation of *β*-catenin, and expression of Snail [146]. In A549 lung adenocarcinoma cells, tunicamycin treatment increased the expression of IL-32 mRNA and the ERS marker GRP78 at both the mRNA and protein levels. A morphological change from a pebble-like shape to an irregular elongated shape accompanied by downregulation of E-cadherin and increased expression of mesenchymal cell markers N-cadherin, vimentin, and ZEB1 was found in IL-32-treated cells. Silencing of IL-32 or treatment with the ERS inhibitor 4-PBA inhibited EMT [147]. Similarly, exposure to tunicamycin or bleomycin changed A549 cell morphology to an elongated fibroblast-like character with a concomitant upregulation of GRP78 and increased expression of N-cadherin, *α*-SMA, and collagen I. The underlying mechanisms of tunicamycin- or bleomycin-induced ERS and EMT are mediated by the upregulation of histone deacetylases HDAC2 and HDAC6 [148].

A direct role of ERS on EMT is also demonstrated in noncancerous alveolar epithelial cells (AECs) induced with ERS activators tunicamycin and thapsigargin or overexpressed mutant surface protein C that causes accumulation of misfolded proteins [149]. Induction of ERS with either of these chemicals increased expression of chaperone GRP78 and spliced XBP1 (XBP1s), which is consistent with EMT characteristics of AECs as observed in the decreased epithelial markers E-cadherin and zonula occludens-1 (ZO-1) and an increase in the myofibroblast marker *α*-SMA with induced fibroblast-like morphology. This effect of ERS-induced EMT is partly mediated through a Src-dependent pathway and may contribute to pulmonary fibrosis pathogenesis. A similar study demonstrated that ERS induction leads to EMT as a potential mechanism for fibrotic remodeling in lungs in which EMT links to crosstalk of numerous pathways including TGF-*β*, Wnt/*β*-catenin, and Src kinase signaling [150]. However, how ERS directly affects EMT in human cancers remains poorly understood.

Notably, EMT is characterized by a transition of polarized to nonpolarized cells. During transition states of EMT, epithelial cells will undergo morphological changes, including loss of cell polarity and cell-cell adhesion while gaining a more mesenchymal and invasive phenotype. Many extrinsic and intrinsic factors can modulate the EMT (Figure 2). As mentioned above, it is explicit that ERS can induce morphological changes and increase cell invasion and metastasis. The dissemination of cancer cells from primary sites to grow in a new microenvironment at secondary metastatic organs requires the migration, extravasation, and invasion of tumor cells into surrounding tissues. These processes need a reorganization of the actin cytoskeleton, cell adhesion and focal attachment, remodeling of ECM, cell contraction, and detachment. Cells undergoing EMT often express a spindle-like morphology with proteolytic ability by activating the release of MMPs to degrade ECM to allow cell movement [151]. In esophageal squamous cell carcinoma (ESCC), overexpression of ATF4, a PERK effector, is involved with increased expression of MMP-2 and MMP-7 to promote invasion and metastasis of ESCC *in vitro* and *in vivo*. An increase in ATF4 in ESCC tumors is correlated with advanced clinical stage and lymph node metastasis of patients. Activation of UPR via the IRE1-XBP1 pathway can affect cell migration and invasion by changing cytoskeleton dynamics [152]. In GBM, inactivation of IRE1 activity modified cell migration and adhesion by increased stress fiber formation and enhanced RhoA activity. Silencing IRE1 resulted in the upregulation of the SPARC gene encoding extracellular matrix proteins, increased RhoA activation, and focal adhesion kinase (FAK) phosphorylation [153].

Additionally, selective inhibition of IRE1 RNase activity with inactive variants targeting IRE1 kinase and RNase domains (K599A, Y892A, and K907A) increased tumor invasion and/or neovascularization in a glioblastoma xenograft model [154]. Cells overexpressing these variants adopted a mesenchymal characteristic and upregulated genes encoding matrix proteins involved in invasion, including collagens (COL1A1, COL3A1, and COL5A1) and the collagen crosslinker lysyl oxidase (LOX). In another study, overexpression of LOXL2 in MDA-MB-231 breast cancer cells causes an increase in ER overload. This stress activates the IRE1-XBP1 signaling pathway, which induces EMT through upregulation of EMT-TFs [155]. Moreover, it has been shown that IRE1 serves as a scaffold of kinases and adaptor proteins for remodeling the actin cytoskeleton [33, 156, 157]. The C-terminus of IRE1 was found to physically interact with filamin A, a protein that functions in crosslinking actin filaments, regulating the formation of lamellipodia and filopodia to propagate cell movement [158], and depletion of IRE1 leads to an alteration of cytoskeleton arrangement and impaired migration of MEFs. Mechanistically, IRE1 controls cytoskeleton dynamics by activating Rac1, a small RhoA GTPase, independently of its RNase activity. Another UPR mediator, PERK, which regulates intracellular Ca^2+^ fluxes and ER-plasma membrane contacts, has also been reported to interact with filamin A [159]. Loss of PERK leads to a perturbed actin cytoskeleton, reduced focal adhesions, and impaired cell migration.

A role for ERS in EMT still needs a thorough investigation. Intercommunication of the UPR branches is critical to determine a definite metastatic phenotype. Apart from oncogenic activation, a majority of studies use ER stressors to acutely activate ERS and investigate molecular events involved in regulating the response [160]. Long-term activation of ERS within tumor stroma and surrounding microenvironments has not yet received much attention as it is becoming more apparent that the UPR can regulate the tumor microenvironment [161]. Future research should further explore the impact of ERS and crosstalk of the UPR on cancer and stromal cells to fully decipher mechanisms of UPR-driven tumor progression by reflecting phenotypes under the complex nature of the tumor microenvironment and cell heterogeneity. Furthermore, it would be of interest to identify unexplored EMT-related genes, epigenetic regulators, or posttranslational mechanisms involved. For the latter point, all proteins have to be modified in the ER and posttranslational modifications (PTMs) determine their maturation and functionality. The PTMs of EMT regulators including Snail, ZEB, and bHLH transcription factors that suppress transcription of epithelial marker genes and activate genes associated with the mesenchymal phenotype are widely studied as a regulatory mechanism of the EMT process. However, the direct effect of ERS on the PTMs of several other functional proteins and enzymes linked to EMT has remained unclear. Moreover, PTM regulatory pathways of the UPR sensor proteins controlling cell survival, apoptosis, EMT, and metastasis have not yet been described in detail. Delineating which PTMs are critical for modulating UPR signaling and ER homeostasis to drive EMT might provide a better understanding of how EMT-related genes and proteins are regulated during ERS and how to modulate PTMs to prevent EMT and cancer progression which might open new opportunities for cancer treatments. A complete understanding of ERS modulation combined with targeting the UPR and its downstream effectors might provide an alternative treatment strategy for the efficient prevention of tumor adaptation and progression.

### 6.2. Three UPR Sensors and Their Underlying Molecular Pathways Involved in EMT

Overcoming ERS is achieved by activating primary UPR sensors, IRE1, PERK, and ATF6, with differential downstream signaling to regulate various responses during ERS. An intraluminal chaperone GRP78/BIP acts in concert with enzymes and UPR sensor proteins to relieve protein misfolding or inhibit protein synthesis. However, the persistent activation of ERS and the inability of the ER to clear misfolded proteins lead to apoptotic cell death. Tumor cells with high metabolic demands are prone to nutrient depletion, hypoxia, acidosis, and poor vascularization, which eventually trigger ERS and UPR as a prosurvival mechanism [162]. Adaptation of tumor cells via UPR activation has been described to participate in tumor development by promoting growth, survival, EMT, and metastasis [163]. Under ERS, UPR markers are upregulated in cancers *in vitro* and *in vivo*. As described above, a growing number of studies have recently reported the correlation between differential expression of UPR components and EMT. It is essential to understand the molecular mechanisms of how tumor cells exploit UPR to mediate EMT-driven tumor progression under ERS. More clarification of the signaling pathways is necessary to further identify potential biomarkers derived from the UPR-EMT axis in tumor tissues to serve as a tool to predict prognosis and therapeutic response in cancer patients.

#### 6.2.1. IRE1/XBP

IRE1/XBP1 signaling is activated in malignant tumors and plays numerous roles in tumor growth and aggressiveness [164–166]. Several functional studies have shown that targeting the expression or RNase activity of IRE1 reduces tumor progression by suppressing the XBP1-mediated survival effect on tumor growth. High levels of spliced forms of XBP1 (XBPs) have been correlated with poor prognosis and low survival rate in human tumors [167–169]. For instance, the IRE1-XBP1 pathway is hyperactivated in melanoma, and XBP1s is overexpressed in tumor specimens compared with normal tissues from patients [170]. In colorectal carcinoma (CRC), overexpression of IRE1 promoted the invasive ability of CRC cells and correlated with poor patient survival. In contrast, the knockdown of IRE1 in turn suppressed invasion with increased expression levels of the epithelial marker E-cadherin and decreased expression levels of the mesenchymal marker N-cadherin, indicating the important role of the IRE1-XBP1 pathway in metastasis of CRC through EMT induction [171]. Differential expression levels of XBP1 are found in CRC cell lines and tumor tissues from a subset of CRC patients [172]. Moreover, in hepatocellular carcinoma (HCC), the expression of XBP1s is detected in HCC cell lines and tissue samples, which is correlated with poor prognosis. Invasiveness and metastasis of HCC cells are promoted by activation of EMT with increased levels of Snail, Twist, and vimentin and decreased levels of E-cadherin and *γ*-catenin [173]. Also, in esophageal squamous cell carcinoma (ESCC), XBP1 is overexpressed in both cell lines as well as in clinical tumor samples, correlating with tumor stage, lymph node metastasis, and poor patient outcome. Using *in vitro* and *in vivo* models, it was demonstrated that XBP1 promoted ESCC invasion and metastasis via the upregulation of MMP-9 [174].

In the case of GBM, hyperactivation of the IRE1-XBP1 axis correlates with poor patient survival, high invasiveness, and immune cell infiltration. These aggressiveness properties of GBM may be controlled through IRE1 signaling as the sequencing of the IRE1 gene (ERN1) in GBM samples revealed a somatic A414T mutation correlating with high vascularization and strong XBP1s staining. Overexpression of wild-type IRE1 in primary derived GBM cell lines is associated with increased expression of EMT-related genes and cytokines [136]. Similarly, high levels of XPBs are detected in primary triple-negative breast cancer (TNBC) cell lines. XBP1 regulates TNBC anchorage-independent growth and invasiveness to promote tumorigenicity, tumor progression, and recurrence, a significant role for XBP1 in regulating the HIF1*α* transcriptional program [175]. In addition to splicing of XBP1, IRE1 has also contributed to the degradation of a subset of tumor-suppressive miRNAs in breast cancer, suggesting unexpected roles of IRE1 in tumor initiation and progression [176].

#### 6.2.2. PERK/eIF2*α*

Compared to the IRE1/XBP1 axis, the mechanisms of PERK-mediated EMT are not well described. This arm of the UPR is mostly related to tumor growth and survival by mediating protein synthesis through eIF2*α* phosphorylation and cell death in part through the ATF4/CHOP pathway [177]. Nevertheless, current evidence suggests that PERK/ATF4 signaling drives EMT by promoting cancer cell migration and invasion [141, 178]. In breast cancer, the activation of PERK signaling correlates with the initiation of EMT and metastasis [179]. The transcription factor CREB3L1 was identified as functioning downstream of PERK to promote EMT and metastatic functions. Activation of this UPR branch may induce EMT under hypoxic stress where the PERK/ATF4/LAMP3 arm of the UPR increases breast cancer cell migration and invasion induced under moderately hypoxic condition (1% O_2_) [141]. PERK is also responsible for the constitutive activation of Nrf2 in EMT dedifferentiated breast epithelial cells, leading to the activation of a multidrug-resistant (MDR) mechanism and insensitivity to chemotherapy [180]. In pancreatic cancer cells, induction of acute ERS with thapsigargin activated PERK phosphorylation, accompanied by a decrease of EMT epithelial markers E-cadherin and ZO-1 and an increase of mesenchymal regulators Snail1, Slug, and ZEB1 [181].

#### 6.2.3. ATF6

Expression of ATF6 mRNA and protein levels is detected in cancer cell lines and tumor samples [182–184]. However, the underlying role for ATF6 in tumorigenesis is largely undefined. Although the UPR has been described as an upstream regulator of EMT, it has also been reported that EMT is a causative process that can activate ERS and UPR signaling. In CRC cells, the induction of ZEB1, which is a major EMT regulator, is a prerequisite for activating ERS. Under conditions of hypoxia or serum starvation, the knockdown of ZEB1 displayed less ERS response as indicated by a decreased expression of GRP78 [185]. Interestingly, Feng et al. revealed that breast cancer cells undergoing EMT are more sensitive to ER stressors as a result of increased ER loads due to the synthesis and secretion of promigratory ECM components and expression of genes encoding ECM proteins [178]. In this context, EMT cells and tumor tissues show selective activation of the PERK-eIF2*α*-ATF4 UPR signaling axis required to promote migration and progression. The undetectable activation of other UPR branches, IRE1 or ATF6, was also observed during EMT.

Nonetheless, it is interesting that activation of UPR and EMT can also occur simultaneously through ROS- and c-Src kinase-dependent pathways in proximal tubular epithelial cells treated with albumin [186]. Inhibiting ER function with related UPR signaling in cells that have undergone EMT may be a feasible approach for cancer treatment. Individual UPR pathways may be interconnected to efficiently drive EMT, but this might be dependent on tumor type and/or ER stressors. It is unknown if signal transduction pathways and/or transcription factors related to EMT and UPR form a feedback loop during tumor progression. Therefore, it is necessary to fully elucidate the interrelationship between UPR signaling and EMT signaling to better understand the mechanism of tumor aggressiveness and support anticancer drug development.

## 7. Conclusion and Future Perspective

Activation of GPCRs and ERS has been shown to promote tumor growth, EMT, and metastasis in multiple neoplasms. This suggests that modulating GPCR-ERS pathways might represent a novel cancer therapeutic option. Although GPCRs are potential targets for cancer therapy, targeting the receptors remains a challenge for anticancer drug development and clinical use. GPCR pathways typically involve physiological functions and human diseases, including cancers, by specific binding of ligands to the receptors, activation of kinases and Rho-GTPases, and eliciting crosstalk with intracellular molecules through various G protein subunits. The signaling complexes of GPCRs are dynamic, and the fact that GPCRs control a wide range of downstream signaling pathways makes it difficult to develop a selective GPCR inhibitor without disturbing normal physiology. Orphan GPCRs with unidentified endogenous ligands also impede the discovery of newly druggable GPCRs. Using high-throughput screening technology, pharmacological manipulation, and genome editing techniques might provide a potential approach for identifying new ligands and modulating GPCR functions in cancer-specific contexts. Importantly, for cancer progression, it remains unclear which G protein subunits, binding partners, and effectors of GPCRs have a predominant impact on EMT and metastasis and how to specifically block their activities without producing undesirable side effects on normal cells. Identifying components of GPCR complexes and interacting molecules mostly relevant to cancer migration and invasion and understanding molecular mechanisms underlying the activation of GPCRs in different tumors will provide more insights and opportunity for the rational design of selective therapeutic strategies against cancer.

A hallmark of cancer is a disruption of ER homeostasis, and it is established that cancer cells can display ERS and UPR activation due to increased metabolic demands, which contribute to the acquisition of EMT to promote tumor progression. This adaptive ability allows cancer cells to disseminate from primary sites and colonize distant organs with an increased likelihood of resistance to therapies. A number of previously published studies demonstrate the contribution of ERS modifiers and the UPR signaling in oncogenesis by promoting tumor growth and survival. Emerging evidence has currently shown that the UPR pathways are also greatly involved in tumor aggressiveness through induction of EMT. Therefore, it is important to consider how various ER stressors convey signals to transform epithelial cells into mesenchymal-like features. The transition between EMT and MET is determined by the expression of EMT-TFs and the tumor microenvironment and other factors. The linkage between UPR and EMT is still debated, and further studies are needed to address essential questions regarding mechanisms that promote EMT by using new molecular approaches and preclinical models. For example, how does ERS induce EMT-MET or cancer reprogramming? Does this involve affecting the tumor microenvironment or reciprocal regulation? What would be the key determinants for this conversion? What intracellular machinery is necessary, and what are the responsible pathways?

## Figures and Tables

**Table 1:** Alteration of GPCR expression in cancer.

GPCR mRNA expression in cancer	Source of mRNA data	Cancer type studied
GPCRs frequently upregulated in most cancer types: FPR3, F2RL1, GPR160, GPR143, P2RY6, APLNR, OPN3CXCR3, CCR1, FZD2, LPAR5, CELSR3, ADORA2B, CCR5, PTAFR, GRP39, F2R, C3AR1, GP5CA, and CELSR1 GPCRs frequently downregulated in most cancer types: GABBR1, GRP146, ACKR1, MRGPRF, LTB4R, SIPR1, ADGRA2, PTGIR, FZD4, ADGRL4, LPAR1, EDNRB, GRP4, MC1R, ADGRD1, ADGRF5, VIPR1, ACKR3, LPAR6, and ADORA2A	TCGA data for tumors vs. GTEx database for normal tissue expression	Adrenocortical cancer, bladder cancer, breast cancer, cervical cancer, colon adenocarcinoma, esophageal cancer, kidney cancer, liver hepatocellular carcinoma, lung cancer, ovarian cancer, pancreatic cancer, prostate adenocarcinoma, skin cutaneous melanoma, stomach adenocarcinoma, testicular cancer, thyroid cancer, uterine carcinosarcoma [24]

GPCRs frequently upregulated in B-CLL cell lines: CXCR4, EBI2, CCR7, ADRB2, PTGER4, GABBR1, CNR2, CELSR1, and LPAR5 GPCRs frequently upregulated in breast cancer cell lines: FZD6, GPR126, P2RY11, CD97, GPRC5B, FZD1, GPR153, OXTR, BAI2, FZD4, LPHN2, GPR161, FZD2, FZD7, TBXA2R, F2R, GABBR1, MC1R, ADORA2B, GPR125, GPR135, OPN3, and EDG3 GPCRs frequently upregulated in colon cancer cell lines: F2RL1, ADORA2B, VIPR1, OXER1, LPAR2, GPR160, and GPRC5A GPCRs frequently upregulated in pancreatic ductal adenocarcinoma tumors: GPRC5A and GPR68	(1) TaqMan GPCR arrays (2) TCGA data for tumors vs. GTEx database for normal tissue expression (3) EBI database	B-cell chronic lymphocytic leukemia cell lines, colon cancer cell lines, triple-negative breast cancer cell lines, pancreatic ductal adenocarcinoma tumors [187]

PAR (1-4) family of GPCRs has high expression in cancer, with frequent upregulation of PAR1 and PAR3 in multiple cancers	Protease-activated receptors in TCGA data for tumors vs. GTEx database for normal tissue expression	Pancreatic adenocarcinoma, esophageal carcinoma, stomach adenocarcinoma, breast invasive carcinoma, head and neck squamous carcinoma, and kidney renal clear cell carcinoma [188]

More than 1.5-fold expression was observed for ~18 GPCRs in prostate cancer and ~30 in breast cancer. AGTR1, F2R, and FPR1 were predicted as targets for prostate cancer and CCR7, CXCR3, GPR18, GPR19, GPR37, GPR171, and GPR171 for breast cancer	Analysis was done using the GEO repository	Prostate cancer and breast cancer [189]

**Table 2:** Oncogenic GPCRs in cancer.

	Phenotypes due to upregulation	Cancer type studied
GPCRs reported with oncogenic functions		
Leucine-rich repeat-containing GPCR4 (LGR4)	Epithelial-mesenchymal transition and cancer stemness [17]	Breast cancer
Protease-activated receptor 1 (PAR1)	Tumor growth, epithelial-mesenchymal transition, invasion, metastasis, and cancer stemness [190–192]	
G protein-coupled receptor GPR19	Mesenchymal-epithelial transition (MET) [14]	
G protein-coupled estrogen receptor-1 (GPER)	Cancer stemness [193]	
G protein-coupled receptor GPR55	Metastasis [194]	
G protein-coupled receptor 56 (GPR56)	Cell growth and epithelial-mesenchymal transition [16]	Colorectal cancer
G protein-coupled chemoattractant receptor formylpeptide receptor-2 (FPR2)	Migration and proliferation, tumor growth [195]	
Lysophosphatidic acid receptor 6 (LPAR6)	*In vivo* tumor growth [196]	Liver cancer
CC-chemokine receptor 10 (CCR10)	Proliferation [197]	
G protein-coupled receptor GPR55	Proliferation and tumor growth [198]	Pancreatic cancer
Leucine-rich repeat-containing G protein-coupled receptor 4 (LGR4)	Epithelial-mesenchymal transition and metastasis [199]	Prostate cancer
Lysophosphatidic acid receptor 1 (LPAR1)	Cell proliferation [200, 201]	
G protein-coupled receptor family C group 6 member A (GPRC6A)	Tumor migration and invasion [202]	
GPCR ligands as oncogenes and their cognate receptors		
R-spondins-G-coupled receptors LGR4/5/6	Cell proliferation [203]	Breast cancer, Colon cancer
Estrogen-GPER1	Proliferation, migration, and invasion [204]	Breast cancer
LPA-LPA receptors	Cell proliferation [205], migration and invasion [206], migration and metastasis [207, 208], cell motility and invasion [209], metabolic shift and cancer-associated fibroblast phenotype [210], metastatic	Colon cancer, gastric cancer, ovarian cancer
Angiotensin II	Invasion and migration [211], cancer stemness [212], proliferation and adhesion potential [213], migration and invasion [214], proliferation, migration, and tumor growth [215]	Head and neck cancer, lung cancer, endometrial cancer, gastric cancer

**Table 3:** Alterations in G protein and GRKs in cancer.

	Cancer type studied
Alteration in G proteins	
Mutations in G*α* protein (GNAS, GNAQ, and GNA11) [27]	Appendiceal tumors (20%), breast cancer (16.40%), colorectal cancer (12.7%), lung cancer (10.9%), hematologic cancers (9.10%), melanoma (excluding ocular) (7.3%), and gastrointestinal cancer (excluding appendiceal and colorectal) (7.3%)
Mutations in G*β* protein (GNB1 and GNB2) [28]	Bladder cancer, breast cancer, hematopoietic and lymphoid malignancies, head and neck cancer, kidney cancer, liver cancer, lung cancer, ovarian cancer, pancreatic cancer, skin cancer, stomach cancer, uterine cancer
GNAL [4]	Endometrial cancer, large intestine cancer, liver cancer, lung cancer, ovarian cancer
GNG12 [4]	Lung cancer
GNA13 [4]	Breast cancer, endometrial cancer, hematopoietic and lymphoid malignancies, kidney cancer, large intestine cancer, lung cancer
GNA14 [4]	Endometrial cancer, kidney cancer, large intestine cancer, lung cancer, ovarian cancer, prostate cancer, skin cancer
Alterations in GRKs	
GRK2	Medulloblastoma [216], gallbladder cancer [217]
GRK3	Prostate cancer [218, 219], colon cancer [220], breast cancer [221]
GRK4	Breast cancer [222]
GRK5	Breast cancer [223], non-small-cell lung cancer [224], prostate cancer [225, 226], renal cell carcinoma [227], glioblastoma [228]
GRK6	Colorectal carcinoma [229], papillary thyroid carcinoma [230], medulloblastoma [231]

## Data Availability

The data used to support the findings of this study are available from the corresponding author upon request.
